# Computer-aided endoscopic diagnostic system modified with hyperspectral imaging for the classification of esophageal neoplasms

**DOI:** 10.3389/fonc.2024.1423405

**Published:** 2024-12-02

**Authors:** Yao-Kuang Wang, Riya Karmakar, Arvind Mukundan, Ting-Chun Men, Yu-Ming Tsao, Song-Cun Lu, I-Chen Wu, Hsiang-Chen Wang

**Affiliations:** ^1^ Graduate Institute of Clinical Medicine, College of Medicine, Kaohsiung Medical University, Kaohsiung, Taiwan; ^2^ Division of Gastroenterology, Department of Internal Medicine, Kaohsiung Medical University Hospital, Kaohsiung Medical University, Kaohsiung, Taiwan; ^3^ Department of Medicine, Faculty of Medicine, College of Medicine, Kaohsiung Medical University, Kaohsiung, Taiwan; ^4^ Department of Mechanical Engineering, National Chung Cheng University, Chiayi, Taiwan; ^5^ Department of Medical Research, Dalin Tzu Chi Hospital, Buddhist Tzu Chi Medical Foundation, Chiayi, Taiwan; ^6^ Technology Development, Hitspectra Intelligent Technology Co., Ltd., Kaohsiung, Taiwan

**Keywords:** Esophageal Cancer, Hyperspectral imaging, Dysplasia, SSD, YOLOv5, Narrow-band imaging

## Abstract

**Introduction:**

The early detection of esophageal cancer is crucial to enhancing patient survival rates, and endoscopy remains the gold standard for identifying esophageal neoplasms. Despite this fact, accurately diagnosing superficial esophageal neoplasms poses a challenge, even for seasoned endoscopists. Recent advancements in computer-aided diagnostic systems, empowered by artificial intelligence (AI), have shown promising results in elevating the diagnostic precision for early-stage esophageal cancer.

**Methods:**

In this study, we expanded upon traditional red–green–blue (RGB) imaging by integrating the YOLO neural network algorithm with hyperspectral imaging (HSI) to evaluate the diagnostic efficacy of this innovative AI system for superficial esophageal neoplasms. A total of 1836 endoscopic images were utilized for model training, which included 858 white-light imaging (WLI) and 978 narrow-band imaging (NBI) samples. These images were categorized into three groups, namely, normal esophagus, esophageal squamous dysplasia, and esophageal squamous cell carcinoma (SCC).

**Results:**

An additional set comprising 257 WLI and 267 NBI images served as the validation dataset to assess diagnostic accuracy. Within the RGB dataset, the diagnostic accuracies of the WLI and NBI systems for classifying images into normal, dysplasia, and SCC categories were 0.83 and 0.82, respectively. Conversely, the HSI dataset yielded higher diagnostic accuracies for the WLI and NBI systems, with scores of 0.90 and 0.89, respectively.

**Conclusion:**

The HSI dataset outperformed the RGB dataset, demonstrating an overall diagnostic accuracy improvement of 8%. Our findings underscored the advantageous impact of incorporating the HSI dataset in model training. Furthermore, the application of HSI in AI-driven image recognition algorithms significantly enhanced the diagnostic accuracy for early esophageal cancer.

## 1 Introduction

Esophageal cancer ranks as the seventh most prevalent cancer and the sixth leading cause of cancer-related deaths worldwide. In 2021, it accounted for 1 in every 18 cancer fatalities (1). Esophageal squamous cell carcinoma (ESCC) is the predominant type of esophageal cancer, constituting over 80% of cases in Asia, Africa, and South America (1, 2). The early detection of superficial esophageal cancer is vital for enhancing patient survival because early-stage diagnoses allow for curative treatments. Notably, nearly 80% of patients with early-diagnosed ESCC have a survival rate extending beyond five years. This rate plummets to below 20% for patients diagnosed at advanced stages (3, 4).

However, in clinical settings, identifying superficial esophageal neoplasms remains a challenge for endoscopists using standard esophagogastroduodenoscopy. Reports indicate a 10%–40% miss rate for early esophageal cancer detection using white-light imaging (WLI) in conventional upper endoscopy (5). Although various enhanced imaging techniques such as narrow-band imaging (NBI) and magnified endoscopy have been introduced to improve early cancer diagnosis, the diagnostic accuracy significantly depends on the endoscopist’s experience and training (6).

The advent of artificial intelligence (AI) and deep-learning algorithms such as convolutional neural networks (CNNs) has led to the creation of several computer-aided systems for the detection and diagnosis of ESCC (7). Notably, Guo et al. implemented the SegNet architecture to devise a computer-assisted diagnosis (CAD) system capable of autonomously identifying precancerous conditions and early ESCC (8). Additionally, the Single Shot MultiBox Detector (SSD) algorithm has been used to develop CAD systems, demonstrating robust diagnostic capabilities in the detection and differentiation of ESCC (9–11). Furthermore, AI frameworks developed using SSD or GoogLeNet have been utilized to ascertain the invasion depth of esophageal cancer (12, 13). The SSD, along with the Bilateral Segmentation Network, has also been applied for the real-time diagnosis of esophageal cancer by using video datasets (14, 15).

Hyperspectral imaging (HSI) is a superior imaging technique that surpasses traditional red–green–blue (RGB) imaging by providing richer information. Its exceptional spectral resolution under 10 nm, along with distinctive spectral signatures for various substances, offers significant advantages in image recognition (16). The clinical utility of HSI in gastrointestinal cancer surgeries has notably increased, aiding in tasks such as detecting anastomotic leaks, identifying optimal anastomotic sites, and delineating colon-cancer margins (17).

Innovative strides have been made with the development of an HSI-based computer-aided diagnosis (CAD) system by Ma et al., which enhances the diagnostic precision for head and neck squamous cell carcinoma on histological slides (18). Similarly, Lindholm et al. adopted an HSI-CNN framework to distinguish between malignant and benign pigmented and non-pigmented skin tumors (19).

Previous research has explored various imaging modalities, but studies on the application of HSI technology in AI systems for esophageal cancer assessment are few (7).

Our research team has developed a CAD system that leverages HSI spectral data and the SSD algorithm for the detection and differentiation of esophageal squamous neoplasms. Our findings indicate that the diagnostic accuracy is enhanced by 5% using HSI spectral data compared with models based on RGB images (20, 21).

While HSI has been used for diagnosing colon, head and neck cancers, and skin tumors, applying it to esophageal cancer poses unique challenges due to the internal location and complex tissue structure. Unlike surface-level cancers, esophageal neoplasms, especially early-stage dysplasia, require precise spectral differentiation. Our innovative approach converts white-light images (WLI) into narrow-band imaging (NBI) similar to the Olympus endoscope using HSI data, enabling the detection of subtle tissue changes. Integrating the YOLOv5 algorithm with HSI enhances diagnostic accuracy, particularly in detecting early dysplasia, offering a significant improvement over existing methods in similar studies. In the current work, our objective was to develop and validate an innovative CAD system by utilizing a deep-learning model known as You Only Look Once (YOLO) in conjunction with HSI technology. Additionally, we assessed its performance relative to models trained on RGB images. Our hypothesis posits that the integration of HSI can enhance the diagnostic capabilities of the CAD system when utilizing CNNs beyond the SSD algorithm. To address the need for enhanced diagnostic precision in esophageal cancer, this study hypothesizes that integrating HSI can improve detection accuracy beyond what is achieved with current imaging methods and AI algorithms. Unlike traditional imaging approaches, HSI captures spectral data across multiple wavelengths, allowing for detailed tissue characterization and identification of subtle differences in tissue composition. Although AI-based diagnostic tools in gastroenterology have shown promising results, they face limitations, such as difficulty in distinguishing early-stage lesions and reliance on narrowband imaging. Many studies have highlighted these limitations, underscoring the need for more advanced imaging modalities. By combining HSI with machine learning, this study aims to overcome these challenges, providing a comprehensive, multi-spectral approach that could improve diagnostic outcomes in esophageal cancer and set a new standard for AI-assisted imaging in gastroenterology.

## 2 Material and methods

### 2.1 Dataset

This study utilized endoscopic images from 16 individuals, comprising 7 patients with esophageal squamous cell carcinoma (ESCC), 9 with squamous dysplasia, and 10 healthy subjects as controls, for neoplastic and normal esophageal imagery. While the sample size of 16 individuals may seem limited, the detailed spectral data captured by hyperspectral imaging (HSI) compensates for this limitation, offering rich diagnostic information per individual. Previous studies using similar sample sizes in HSI research have demonstrated reliable outcomes. Future work will expand the dataset, but this study demonstrates the feasibility of our approach. Pathological assessments were performed to categorize the esophageal neoplasms. Following the removal of images that were unmarked, blurred, or out of focus, a dataset of 1836 images was compiled for model training. This dataset included 858 white-light imaging (WLI) and 978 narrow-band imaging (NBI) pictures. Specifically, the WLI collection contained 219 SCC images, 159 squamous dysplasia images, and 480 normal esophagus images. The NBI set included 222 SCC images, 306 squamous dysplasia images, and 450 normal esophagus images. An additional 257 WLI and 267 NBI images were designated as the test set. The Institutional Review Board of Kaohsiung Medical University Hospital (KMUH) granted ethical approval for this research (KMUHIRB-E(II)-20190376).

### 2.2 Hyperspectral imaging conversion

**Figure 1:**
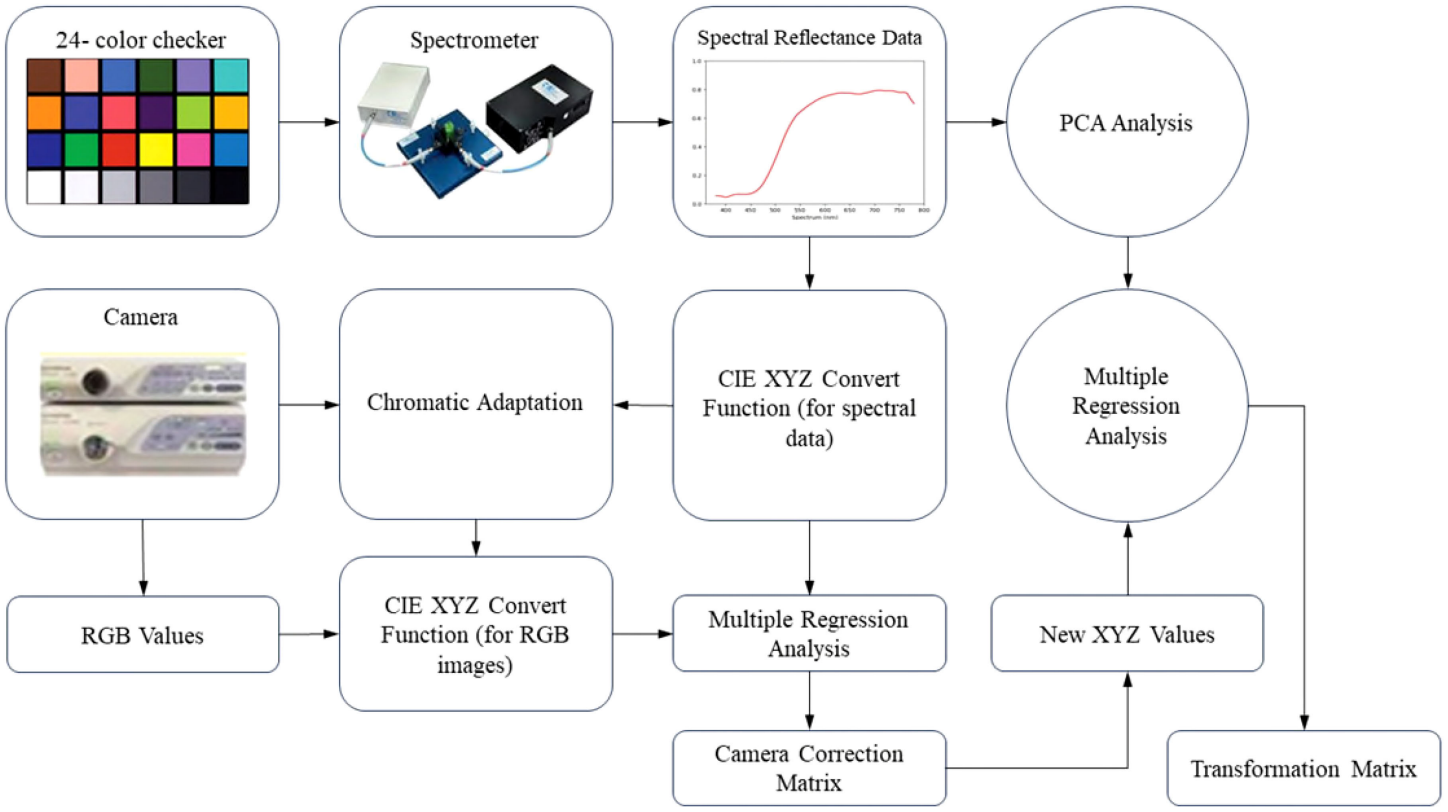
Flow chart of HSI algorithm.


Visible–HSI (VIS-HSI) technique was applied to transform esophageal-cancer images into a spectrum of 401 bands ranging within 380–780 nm. The process of spectrum conversion is depicted in **Figure 1**. To align the spectrometer with the endoscope, a set of standard 24 color blocks was used for calibration reference. The conversion matrix, bridging the endoscope (OLYMPUS EVIS LUCERA CV-260 SL) and the spectrometer (Ocean Optics, QE65000), was derived by capturing images to ascertain chromaticity values and measuring spectra for spectral values. The transition from the sRGB to the XYZ color space within the endoscope’s system was facilitated using Equation 1.

(1) $$\left[\begin{array}{c} X\\ Y\\ Z \end{array}\right]=\left[M_{A}\right]\left[T\right]\left[\begin{array}{c} f\left(R_{sRGB}\right)\\ f\left(G_{sRGB}\right)\\ f\left(B_{sRGB}\right) \end{array}\right]\times 100,\;0\leq \begin{array}{c} R_{sRGB}\\ \begin{array}{c} G_{sRGB}\\ B_{sRGB} \end{array} \end{array}\;\leq 1$$

To comply with the sRGB color-space standards, the RGB values of the endoscopic image must be scaled from a 0–255 range to a 0–1 range. The gamma function was then applied to convert the sRGB values into linear RGB ones. They were further transformed into XYZ values using a conversion matrix, ensuring normalization within the color space. However, the chromatic adaptation transformation matrix was essential during this process to account for discrepancies between the standard D65 white point (*XCW, YCW, ZCW*) of the sRGB space and the actual light source’s white point (*XSW, YSW, ZSW*). The true XYZ values (*XYZ_Endoscopy_*) were obtained with this matrix in the context of the measured light source. In the spectrometer setup, the spectrum of the light source, S(λ), is combined with the XYZ color-matching function. Notably, the luminance value (*Y*) in the *XYZ* space was directly correlated with perceived brightness and was capped at 100. Utilizing the Y value in Equation 2 facilitated the determination of the light-source spectrum’s maximum brightness and brightness ratio (*k*). Equations 3–5 were used to gather reflective spectrum data.


(2) $$\text{k}=100/\int_{380\text{nm}}^{780\text{nm}}\text{S}\left(\text{λ}\right)\bar{\text{y}}\left(\text{λ}\right)\text{dλ}$$

(3) $$\text{X}=\text{k}\int_{380\text{nm}}^{780\text{nm}}\text{S}\left(\text{λ}\right)\text{R}\left(\text{λ}\right)\bar{\text{x}}\left(\text{λ}\right)\text{dλ}$$

(4) $$\text{Y}=\text{k}\int_{380\text{nm}}^{780\text{nm}}\text{S}\left(\text{λ}\right)\text{R}\left(\text{λ}\right)\bar{\text{y}}\left(\text{λ}\right)\text{dλ}$$

(5) $$\text{Z}=\text{k}\int_{380\text{nm}}^{780\text{nm}}\text{S}\left(\text{λ}\right)\text{R}\left(\text{λ}\right)\bar{\text{z}}\left(\text{λ}\right)\text{dλ}$$

Various elements, including nonlinear response, inaccurate color filter separation, dark current, and color shifts, can lead to errors. To address this issue, we calculated a correction coefficient matrix (*C*) through multiple regression analysis (Equation 6). By multiplying the variable V matrix with this correction matrix, we obtained the adjusted X, Y, and Z values (*XYZ_Correct_*).


(6) $$\left[\text{C}\right]=\left[{\text{XYZ}}_{\text{Spectrum}}\right]\times \text{pinv}\left(\left[\text{V}\right]\right)$$

**Figure 2:**
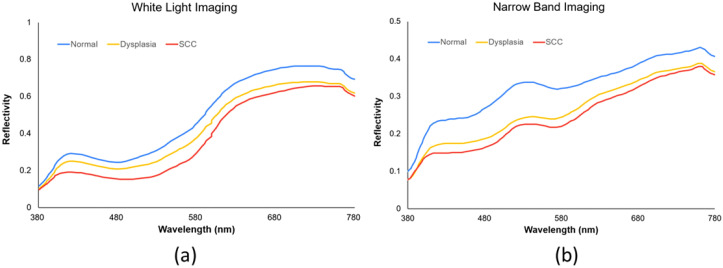
Spectrum distributions of **(A)** WLIs and **(B)** NBIs.

The root-mean-square error (RMSE) was calculated for the *XYZ_Correct_* and *XYZ_Spectrum_* datasets. The WLI and NBI endoscopes exhibited average errors of 1.40 and 2.39, respectively. We derived the transformation matrix (*M*) by correlating the XYZ values of 24 color patches from *XYZ_Correct_* with the spectrometer-measured reflection spectra (*R_Spectrum_*). Principal component analysis (PCA) on the R_Spectrum_ subset identified key components, which were then used to establish the conversion matrix against the XYZ_Correct_ values. We evaluated the RMSE and specific color patch discrepancies by comparing the S_Spectrum_ from the 24-color patch simulation with the R_Spectrum_. The average error for the WLI was found to be 0.057, while it was 0.097 for the NBI. Differences between the SSpectrum and R_Spectrum_ were highlighted by variations in color, with average color errors of 2.85 for WLI and 2.60 for NBI, respectively. The described hyperspectral method for visible light computed and simulated the reflection spectrum from RGB data captured by a single-lens camera. This technology enabled the calculation of RGB values for the full image to produce a hyperspectral image. We analyzed the spectral variances between lesions and non-lesions by marking their locations. As depicted in **Figure 2**, we selected the 415–540 nm band range for PCA due to the significant RMSE among the three categories: SCC, dysplasia, and normal. These categories showed marked differences in reflectivity.

### 2.3 Construction of YOLOv5 system

The YOLOv5 deep CNN model uses a neural network to conduct feature mapping of images, segmenting them into S×S grids (22–25). Herein, it determined the center position offset ( $t_{x},\;t_{y}$ ) and the relative dimensions ( $t_{w},\;t_{h}$ ) for each grid’s prediction and prior frames. The model generated a confidence score for objects, predicted category probabilities, and applied Intersection over Union threshold and non-maximum suppression for final frame selection (26, 27). The architecture incorporated Focus+Cross Stage Partial networks to enhance feature extraction and reduce computational load. Spatial pyramid pooling, feature pyramid networks, and path aggregation networks bolstered the feature mapping across various object sizes. A key advancement in YOLOv5 was the object center alignment within grid cells, improving match accuracy (28). Samples with aspect-ratio changes under fourfold were deemed positive, with two adjacent grids predicting such samples to boost positive detection rates. This adjustment significantly accelerated model convergence, thereby enhancing detection speed.

The YOLOv5 loss function encompassed classification loss, confidence loss, and positioning loss. Classification loss used cross-entropy to gauge the discrepancy between predicted and actual category probabilities. Confidence loss compared the actual and predicted bounding boxes, whereas positioning loss assessed the difference in Complete Intersection over Union values. The weights for these losses were fine tuned, as detailed in Equation 7.


(7) $$L\left(x,c,l,g\right)=\;\frac{1}{N}\left[0.5L_{\text{cls}}\left(p_{\text{gt}},p_{\text{c}}\right)+\;\alpha L_{\text{obj}}\left(l_{\text{gt}},l\right)+0.05L_{\text{box}}\left(l_{\text{gt}},l\right)\right]$$

**Figure 3:**
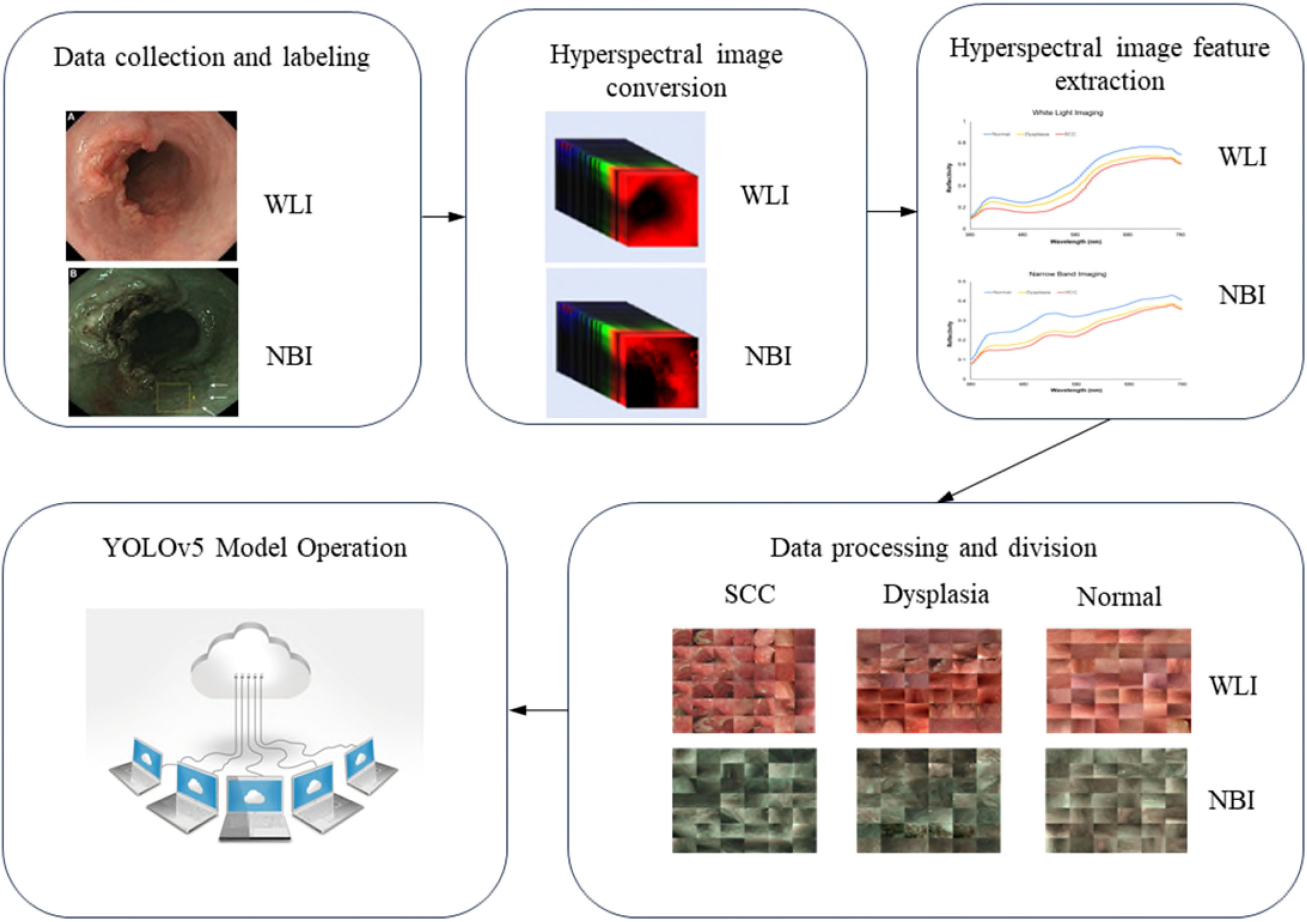
Complete process of the algorithm designed in this study.

Here, N represents the count of matched positive samples, α is the scale-dependent loss function gain index, with the small-to-medium-to-large target gain ratio set at 4:1:0.4, *p*_gt_ denotes the probability for each positive sample category, *p*_c_ for each predicted frame category, *l*_gt_ is the real box’s center location, and *l* denotes the predicted frame’s center location. *L*_cls_, *L*_obj_, and *L*_box_ signify classification, confidence, and localization losses, respectively. An esophageal neoplasm was considered accurately detected if the IOU was ≥0.5. The algorithm’s full workflow is illustrated in **Figure 3**.

**Figure 4:**
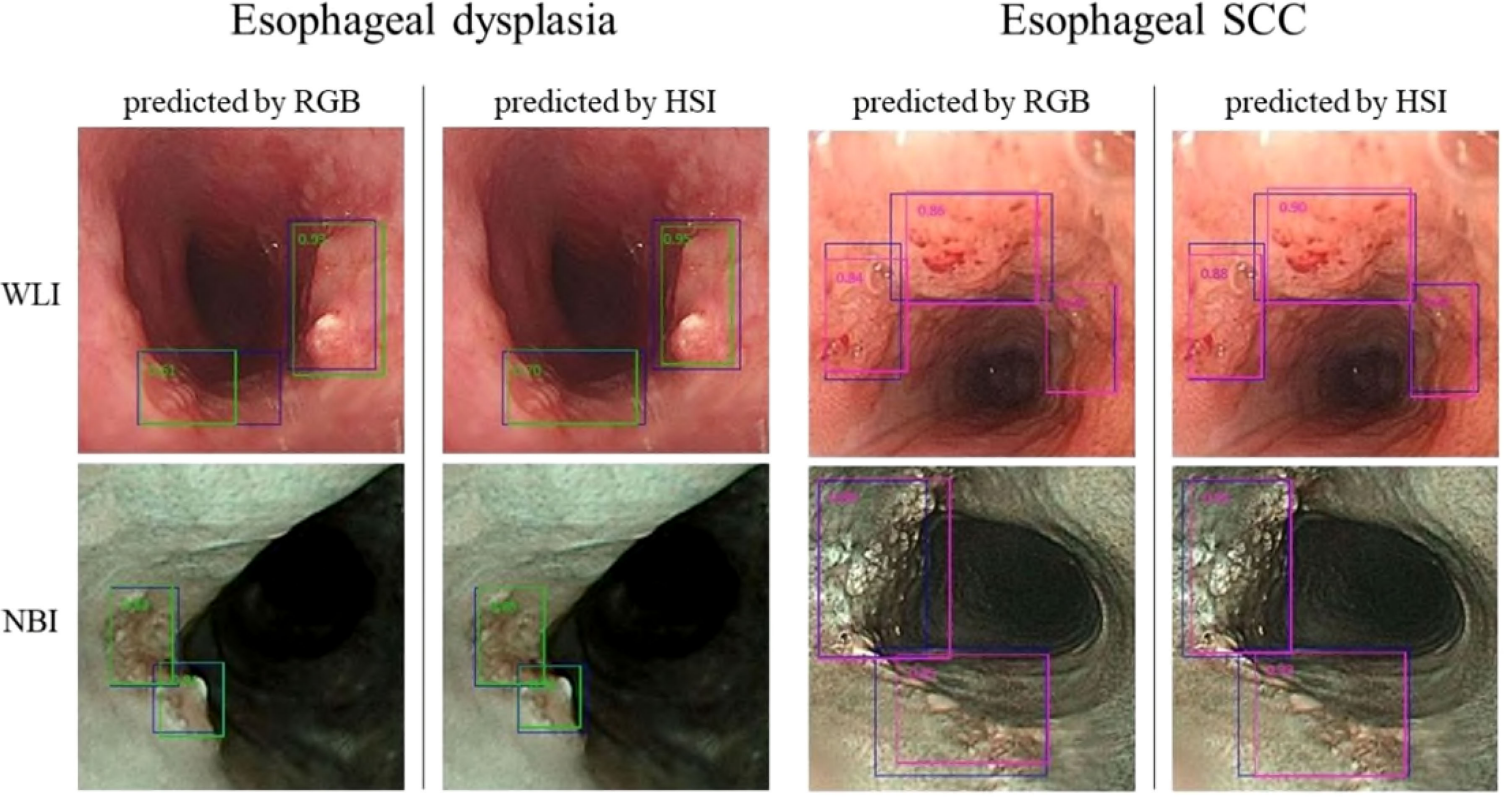
presents the YOLOv5 diagnostic outcomes for the WLI and NBI images of esophageal neoplasms. Blue boxes signify the ground truth. Green-bordered boxes highlight areas identified as esophageal dysplasia, whereas purple-bordered boxes indicate SCC regions. The labels’ numbers indicate the likelihood of an esophageal-neoplasm diagnosis within the box.

In this study, we utilized 524 separate images for the test set, comprising 257 WLI and 267 NBI images, to assess the YOLO combined with the HSI system’s diagnostic efficacy. We crafted four predictive models that integrated RGB and HSI data in the WLI and NBI modalities to gauge diagnostic precision. For visualization, the images were overlaid with a blue ground truth box. The YOLO framework used a green bounding box to denote esophageal squamous dysplasia and a purple box for ESCC identification. The YOLOv5 system’s diagnostic outcomes are depicted in **Figure 4**.

## 3 Results

### 3.1 Diagnosis performance of YOLO for detecting esophageal neoplasms

The system processed each image in 0.05 s. For the RGB-WLI model, it accurately identified 217 out of 294 esophageal-neoplasm frames and correctly diagnosed 293 out of 321 normal esophagus frames. The HSI-WLI model correctly predicted 252 out of 294 neoplasm frames and 292 out of 308 normal frames, noting fewer frames than RGB-WLI due to unsuccessful transformations. By testing with the NBI dataset, the RGB-NBI and HSI-NBI models accurately predicted 335 and 359 neoplasm frames out of 417, respectively. They also correctly identified 250 out of 299 and 252 out of 269 normal frames, respectively. Diagnostic accuracies for esophageal-neoplasm detection were 83% (RGB-WLI), 82% (RGB-NBI), 90% (HSI-WLI), and 89% (HSI-NBI). The HSI models outperformed RGB models in sensitivity, specificity, precision, and F1-score across the WLI and NBI datasets. Overall, HSI models improved neoplasm-detection accuracy by 8% compared with RGB models, as detailed in **Table 1**. The 8% enhancement in diagnostic accuracy attained using HSI is noteworthy, particularly in facilitating the early identification of premalignant lesions, including esophageal dysplasia. The enhancement in accuracy may impact patient outcomes by facilitating earlier and more accurate therapies. Nevertheless, the therapeutic importance of this enhancement must be meticulously evaluated about the additional complexity, expense, and duration necessary for HSI capture and processing. The enhanced detection skills warrant additional investigation of HSI; however, subsequent research should focus on optimizing the imaging and processing workflow to reduce its effect on clinical workflow efficiency. Enhancing these mechanisms can render HSI more feasible in standard clinical practice. Additionally, a comprehensive examination of HSI’s efficacy in identifying premalignant lesions would yield greater insights into its genuine therapeutic significance, particularly in contexts where early diagnosis might result in improved long-term outcomes.”

**Table 1:** Performance of diagnosis using the YOLOv5 system trained by different datasets for diagnosing esophageal neoplasms.

Diagnostic performance					
Dataset	Sensitivity (%)	Specificity (%)	Precision (%)	F1-score (%)	Accuracy (%)
RGB					
**RGB-WLI**	74	91	89	81	83
**RGB-NBI**	80	84	87	84	82
HIS					
**HSI-WLI**	86	94	94	90	90
**HSI-NBI**	86	99	95	91	89

RGB, red-green-blue; WLI, white light imaging; NBI, narrow band imaging; HSI, hyperspectral imaging.

### 3.2 Diagnosis performance of YOLO for classifying esophageal neoplasms

The WLI dataset included 186 frames of actual esophageal SCC and 108 frames of dysplasia for neoplasm-classification accuracy testing. The RGB-WLI and HSI-WLI models used 321 and 308 frames of genuine normal esophagus, respectively, with some data loss in HSI conversion. In the NBI dataset, 188 frames of SCC and 219 frames of dysplasia were tested for diagnostic accuracy. For normal esophagus, 299 and 269 frames were used in the RGB-NBI and HSI-NBI models, respectively. The outcomes for the RGB-WLI, RGB-NBI, HSI-WLI, and HSI-NBI models are detailed in the confusion matrix (**Table 2**).

**Table 2:** Confusion matrix of YOLOv5 system trained by different datasets for the classification of esophageal neoplasms.

	YOLO diagnosis				
True diagnosis	Normal	dysplasia	SCC	Accuracy (%)	Kappa
RGB					
RGB-WLI					
**Normal**	293	18	10		
**Dysplasia**	32	76	0	83	0.71
**SCC**	45	0	141		
RGB-NBI					
**Normal**	250	38	11		
**Dysplasia**	45	174	0	84	0.72
**SCC**	37	0	161		
HSI					
HSI-WLI					
**Normal**	292	7	9		
**Dysplasia**	17	91	0	90	0.84
**SCC**	25	0	161		
HSI-NBI					
**Normal**	252	9	8		
**Dysplasia**	32	185	2	91	0.83
**SCC**	26	0	172		

SCC, squamous cell carcinoma; RGB, red-green-blue; WLI, white light imaging; NBI, narrow band imaging; HSI, hyperspectral imaging.

The RGB-WLI and HSI-WLI models achieved diagnostic accuracies of 83% and 90%, with Kappa values of 0.71 and 0.92, respectively (**Table 3**). Similarly, the RGB-NBI and HSI-NBI models recorded accuracies of 82% and 89%, and Kappa values of 0.72 and 0.83, respectively. The HSI model improved neoplasm classification accuracy by 8% compared with the RGB model (**Table 2**). Across the WLI and NBI datasets, the HSI model demonstrated superior diagnostic accuracy and Kappa values (**Table 3**). Our system also showed enhanced sensitivity, precision, and F1-scores for SCC compared with esophageal dysplasia in both models. Notably, the NBI system outperformed the WLI system in diagnosing esophageal dysplasia and SCC in terms of sensitivity, precision, and F1-score (**Table 3**).

**Table 3:** Diagnosis performance of YOLOv5 system trained by different datasets for classifying esophageal neoplasms.

		Sensitivity (%)	Specificity (%)	Precision (%)	F1-score (%)	Accuracy (%)	Kappa
**RGB**	WLI						
	**Dysplasia**	70	86	81	75	83	0.71
	**SCC**	76	86	93	84		
	NBI						
	**Dysplasia**	79	83	82	81	82	0.72
	**SCC**	81	82	94	87		
**HSI**	WLI						
	**Dysplasia**	84	92	93	88	90	0.92
	**SCC**	87	92	95	90		
	NBI						
	**Dysplasia**	84	91	95	90	89	0.83
	**SCC**	87	90	95	91		

SCC, squamous cell carcinoma; RGB, red-green-blue; WLI, white light imaging; NBI, narrow band imaging; HSI, hyperspectral imaging.

## 4 Discussion

The prompt detection of esophageal cancer is vital for enhancing patient survival, with endoscopy being the most precise method (29). However, the accuracy for early-stage and superficial neoplasms depends heavily on the endoscopist’s skill. Recent CAD systems leveraging diverse AI algorithms have shown promise in boosting early cancer-detection accuracy (7). Historically, WLI and NBI images have been the primary types used in model training, with limited use of magnified NBI and blue-laser imaging. Various CNN architectures like SSD, ResNet, SegNet, ResNet/U-Net, YOLOv3, and Grad-CAM have been used (30). This study introduced hyperspectral technology alongside traditional RGB imaging to explore HSI’s impact on CAD systems. Our findings indicated that HSI outperformed RGB in the detection and classification of esophageal neoplasms, enhancing diagnostic accuracy by 8%. HSI data offered a three-dimensional dataset, enabling more feature extraction than RGB’s three bands. It extended beyond the visible spectrum, allowing the analysis of characteristics typically imperceptible to the human eye. By capturing the electromagnetic spectrum across numerous narrow wavelengths, HSI significantly improved resolution. Consequently, HSI-WLI and HSI-NBI algorithms yielded superior results over their RGB counterparts (16, 31–33).

Hyperspectral imaging (HSI) technology has found widespread use in distinguishing lesions from normal tissue across various medical domains, including cervical cancer, skin cancer, diabetic foot, and cutaneous wounds (31). However, its application in gastroenterology remains in its early stages, with most research focusing on surgical assistance, including tasks such as anatomy identification, bowel ischemia detection, gastric cancer identification, and pathological support (32). Notably, the utilization of HSI for diagnosing esophageal cancer is still limited. In a study by Maktabi et al., HSI combined with classification algorithms is used for the automatic detection of esophageal cancer (squamous cell carcinoma and adenocarcinoma) in resected tissue samples from 11 patients. The resulting sensitivity and specificity for cancerous tissue are 63% and 69%, respectively (34). Maktabi et al. used HSI technology alongside a machine-learning method (multi-layer perceptron) to differentiate between esophageal adenocarcinoma and squamous epithelium in hematoxylin and eosin-stained specimens. The accuracy rates for esophageal adenocarcinoma and squamous epithelium are 78% and 80%, respectively. Notably, HSI demonstrates superior accuracy compared with the results obtained using RGB data in their study (35). In the current work, we leveraged HSI techniques in conjunction with the YOLOv5 algorithm for diagnosing esophageal neoplasms from endoscopic images. Our findings reaffirmed the beneficial role of HSI in endoscopic image recognition for esophageal neoplasms. Specifically, the diagnostic accuracy of our HSI system improved by 8% compared with the RGB system. Our group has previously explored HSI technology alongside the SSD algorithm for diagnosing esophageal neoplasms. In this case, the HSI-SSD system exhibits a 5% improvement in diagnostic accuracy compared with the RGB-SSD system (20). Recognizing that different algorithms may yield varying effects on the image recognition of HSI data is important. It emphasizes the need for further studies to validate these observations.

Our study further revealed a correlation between malignancy severity and AI diagnosis performance. The YOLOv5 system displayed higher sensitivity for SCC than dysplasia, echoing findings from a prior SSD system study. In the RGB dataset, sensitivities for dysplasia and SCC were 70% and 76% in the RGB-WLI model and 76% and 81% in the RGB-NBI model, respectively. NBI outperformed WLI in diagnosing both conditions (**Table 3**). In the HSI dataset, sensitivities for SCC remained higher than those for dysplasia, i.e., 84% and 87% in the HSI-WLI and HSI-NBI models. However, NBI did not enhance diagnostic capabilities over WLI in the HSI dataset. This finding may be due to the selection of the 415–540 nm band for feature extraction and PCA given the high RMSE among SCC, dysplasia, and normal tissue (36). The original NBI also used the 415–540 nm band, providing high-contrast vascular images for neoplasm detection. Thus, the spectral differences between HSI-WLI and HSI-NBI were minimal, suggesting limited additional feature-extraction benefits from NBI in the HSI dataset.

Our system demonstrated high specificity and precision in identifying esophageal neoplasms; however, sensitivity remained below the optimal levels. A meta-analysis of 24 AI studies reported pooled sensitivity and specificity rates of 91.2% and 80% for ESCC diagnosis (37). The limited sensitivity in our case may stem from a smaller training image set, insufficient for achieving 90% sensitivity. Unlike most studies that categorize lesions as cancerous or non-cancerous, we included a third category, that is, dysplasia, which is crucial to early intervention and survival improvement. However, the impact of incorporating dysplasia in training is unclear because it represents an intermediary stage rather than a distinct entity, which can affect AI performance. This finding suggested a need for future research to refine the algorithm. False negatives were often due to lesions in the esophagus’s shadowed regions, indicating a need for more diverse training images. Additionally, discrepancies between AI-selected frames and manual lesion labeling affected model convergence and diagnostic accuracy. Despite the laborious and time-intensive nature of developing spectral imaging sensors and data analysis methodologies, the information acquired through spectral imaging techniques allows for a clear examination of the characteristics of various tissues and organs in both healthy and diseased subjects, which has not been feasible for direct investigation previously (38). Spectral unmixing and other image processing techniques utilized on hyperspectral data uncover nuanced color and texture variations not observable in conventional microscope images, hence enhancing the pathology of biological specimens, therefore the same method can also be applied to the pathological slides (39).

Our study has several limitations. First, the sample size of the images used was small, and the limited number of training images compared with that in previous studies may have influenced the diagnosis performance of our system. Second, we exclusively utilized static images from standard endoscopes (specifically, the GIF-Q260 and EVIS LUCERA CV-260/CLV-260 systems by Olympus Medical Systems, Co., Ltd., Tokyo, Japan). Consequently, the applicability of our system remained restricted to these conditions. Whether incorporating video images or magnified endoscopy can enhance our system’s diagnosis performance remains uncertain. Lastly, although our study successfully improved the diagnostic accuracy of the CAD system by integrating HSI, the time-consuming conversion process still requires upgrading for practical clinical use. In future studies, there are plans to explore multi-modal imaging techniques that combine HSI with other methods, such as optical coherence tomography (OCT) or ultrasound, to overcome the depth limitation and provide more comprehensive tissue analysis. Although HSI offers significant potential for improving esophageal neoplasm detection, several practical barriers exist, including high hardware costs, complexity in data interpretation, and the need for clinician training. Reducing costs through technological innovation, integrating AI for automated data interpretation, and providing standardized training programs can help address these challenges. Furthermore, conducting clinical pilot studies will aid in validating the system and accelerating regulatory approvals, facilitating smoother integration of HSI into routine clinical practice.

## 5 Conclusion

Our study demonstrated that the HSI-CAD system outperformed the conventional RGB-CAD system in detecting and classifying esophageal neoplasms. Specifically, our system achieved an 8% improvement in diagnostic accuracy. To enhance our system further, additional HSI data should be incorporated. Future investigations on the potential impact of HSI data on other CAD systems are also necessary.

## Data Availability

The original contributions presented in the study are included in the article/supplementary material. Further inquiries can be directed to the corresponding authors.
