# Supplementary material for: Computer-aided endoscopic diagnostic system modified with hyperspectral imaging for the classification of esophageal neoplasms
Source: Front Oncol. 2024 Dec 2;14:1423405. doi: 10.3389/fonc.2024.1423405 (PMC11646837; doi:10.3389/fonc.2024.1423405)
Supplement: Supplementary file 1 [file DataSheet1.docx]

**Computer-Aided Endoscopic Diagnostic System Modified with Hyperspectral Imaging for the Classification of Esophageal Neoplasms**

**Yao-Kuang Wang ^1,2,3^, Riya Karmakar ^4^, Arvind Mukundan ^4^, Ting-Chun Men ^4^, Yu-Ming Tsao ^4^, Song-Cun Lu ^4^, I-Chen Wu ^2,3,∗^, Hsiang-Chen Wang ^4,5,6,∗^**

**1 Graduate Institute of Clinical Medicine, College of Medicine, Kaohsiung Medical University, No.100, Tzyou 1st Rd., Sanmin Dist., Kaohsiung City 80756, Taiwan**

**2 Division of Gastroenterology, Department of Internal Medicine, Kaohsiung Medical University Hospital, Kaohsiung Medical University, No.100, Tzyou 1st Rd., Sanmin Dist., Kaohsiung City 80756, Taiwan; fedwang@gmail.com (Y.-K.W.)**

**3 Department of Medicine, Faculty of Medicine, College of Medicine, Kaohsiung Medical University, No.100, Tzyou 1st Rd., Sanmin Dist., Kaohsiung City 80756, Taiwan**

**4 Department of Mechanical Engineering, National Chung Cheng University, 168, University Rd., Min Hsiung, Chia Yi 62102, Taiwan; karmakarriya345@gmail.com (R.K.); d09420003@ccu.edu.tw (A.M.); d09420002@ccu.edu.tw (Y.-M.T.); asd19970810@gmail.com (T.-C.M.); admsclu@ccu.edu.tw (S.-C.L.)**

**5 Department of Medical Research, Dalin Tzu Chi Hospital, Buddhist Tzu Chi Medical Foundation, No. 2, Minsheng Road, Dalin, Chiayi, 62247 Taiwan**

**6 Director of Technology Development, Hitspectra Intelligent Technology Co., Ltd., 8F.11-1, No. 25, Chenggong 2nd Rd., Qianzhen Dist., Kaohsiung City 80661, Taiwan**

*** Correspondence: minicawu@gmail.com (I.-C.W.) and hcwang@ccu.edu.tw (H.-C.W.)**

**Abstract:** This article provides the supplementary information for the article object detection combined with hyperspectral imaging to classify and detect early esophageal cancer. Section 1 gives an overview of the basic YOLO model while the second section describes the Conv Mods. Section 3 provides the overview of the Focus functions and Section 4 describes the C3 module. SPP module description is given in Section 5 while the final section gives a brief overview of the FPN and PANet Architecture.

**Keywords:** White light imaging, narrow band imaging, hyperspectral imaging, YOLOv5, esophageal cancer.

1. YOLO basic detection method

As shown in Figure S1 (a), the image is cut into S×S grids as the input layer through the neural network. Then x, y, w, h, confidence scores are predicted as shown in Figure 1(b). The predictions box is generated with the grid category as shown in Figure S1(c) and finally boxes are filtered out via maximal NMS as shown in Figure S1(d). In the YOLOv5 neural network architecture, backbone adopts the Focus+CSP (cross stage partial) architecture to optimize the neural network, improve the feature extraction capability of the convolutional neural network (CNN), reduce the amount of computation, and use SPP (spatial pyramid pooling), FPN (feature pyramid networks) and PAN (path aggregation network) structures enhance feature maps of different sizes of target feature information. The overall architecture is shown in Figure S2. The area circled by the red box uses three detectors for large, medium and small objects, which are 1/8 times down sampling to detect small objects, 1/16 times down sampling to detect medium objects, and 1/32 down sampling to detect large objects.

2. Conv Mods

The main function of the Conv module is to use convolution to extract image features or to change the size of the feature map through convolution to enable feature fusion (Concat), as shown in Figure S3.

3. Focus module

The function of the focus module is to integrate the length and width information into the channel dimension through the slice operation This method approximates images of different sizes for feature fusion, but only uses one convolutional layer to optimize the feature extraction capability.

4. C3 module

The C3 module modifies the network structure based on the concept of CSP (cross stage partial) and divides the input into two parts. After the output of the designed network structure is completed, it directly performs feature fusion with the other divided data (concat). This method can keep the features of the base layer as much as possible. It can effectively alleviate the problem that the image information in the back end of the convolution layer has lost due to the poor process of feature extraction. The backpropagation cannot be optimized while the amount of computation is reduced, as shown in Figure S5.

5. SPP module


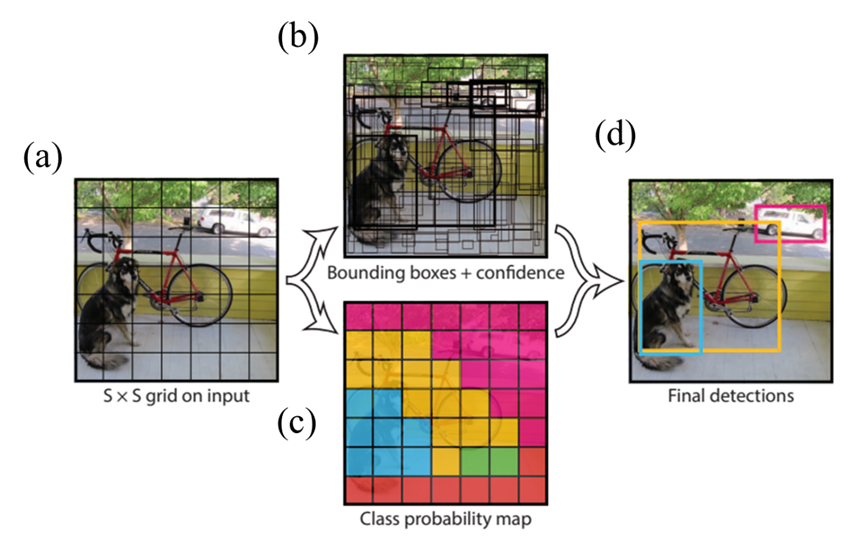
The SPP module performs feature fusion (concat) on the feature map through three maximum pooling layers of different sizes and a 1×1 convolutional layer. This method can enhance the target feature information of different sizes of the feature map, as shown in Figure S6.

Figure S1. Basic detection method of YOLO [1]

6. FPN and PANet Architecture

FPN is the feature fusion of the shallow feature map and the down sampled feature map to improve the detection ability of small targets. PANet is the feature fusion of the down sampled feature map and the up sampled feature map. The main purpose of both is It is combined with the feature map of the shallower layer to enhance the feature information of the target of different sizes in the feature map.

Figure S1. Basic detection method of YOLO [1]


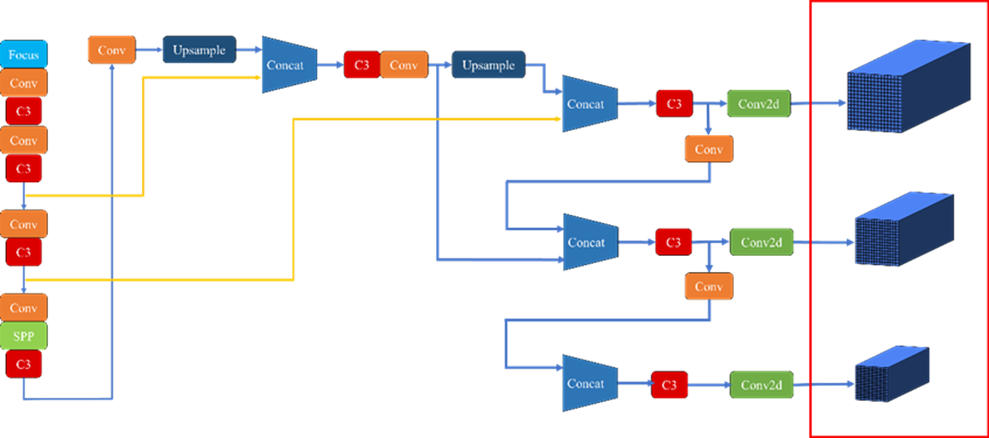
Figure S2. YOLOv5 model architecture


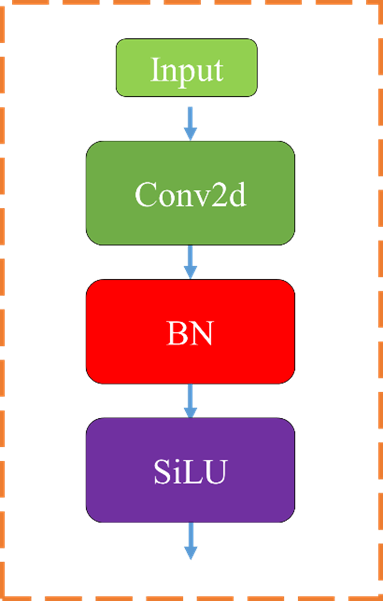


Figure S3. Schematic diagram of Conv module


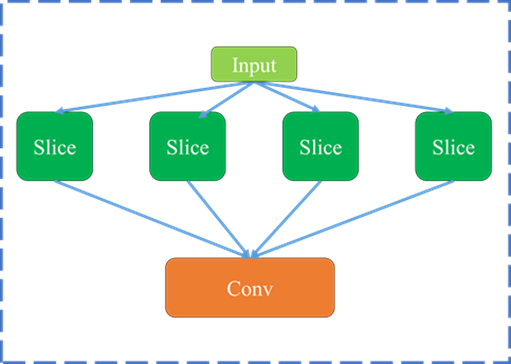


Figure S4. Schematic diagram of Focus module


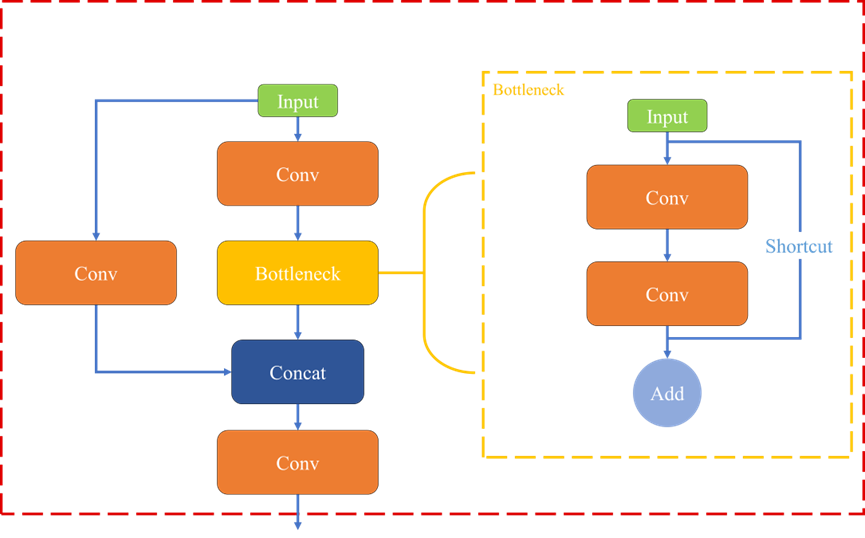


Figure S5. Schematic diagram of C3 module.


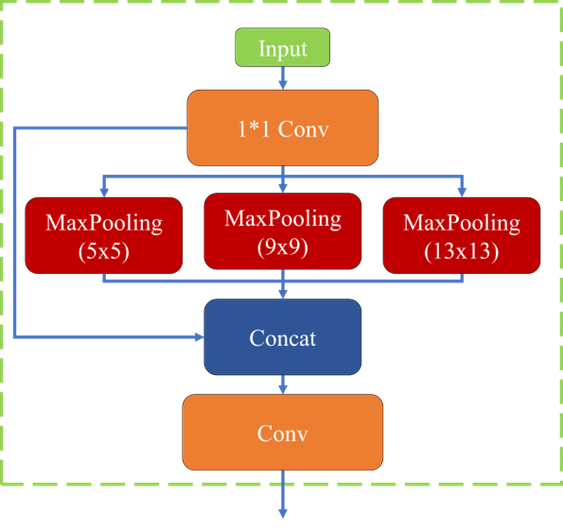


Figure S6. SPP module structure


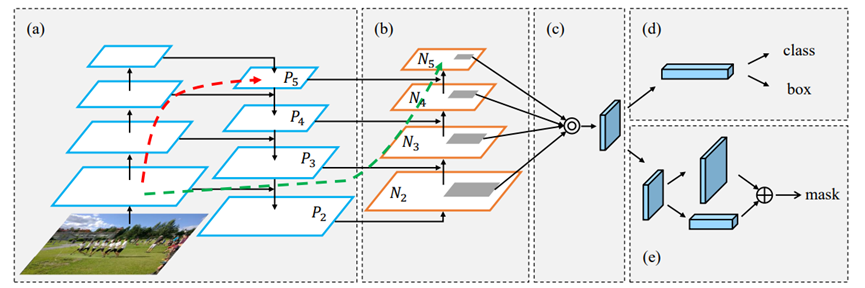


Figure S7. Schematic diagram of FPN and PANet [2].

7 Instrument Specification

The visible hyperspectral imaging (VIS-HSI) used in this study is calculated by using the images taken by a single-lens camera (Nikon D5200) combined with the visible hyperspectral algorithm (VIS-HSA). The wavelength range is from 380 nm to 780 nm, and the spectral resolution is up to 1 nm. The detailed instrument specifications used in this study are mentioned in Table 1.

|  | Specification | Resolution | Components | Bilateral |
| --- | --- | --- | --- | --- |
| Visible Light Camera | Nikon D5200 | 6000*4000 | CMOS | 380-780nm |
| Spectrometer | Ocean Optics QE65000 | 1mm | CCD | 200-1100nm |

Table S1. Instrument Specification.

The individual conversion formulas to convert the 24-colour patch image and 24 colour patch reflectance spectrum data to XYZ colour space are as follows

On the camera side: convert sRGB color gamut space to XYZ color gamut space

$\left[ \begin{aligned} X \\ Y \\ Z \end{aligned} \right]=[M_{A}]\left[ T \right]\left[ \begin{aligned} f\left( R_{sRGB} \right) \\ f\left( G_{sRGB} \right) \\ f(B_{sRGB}) \end{aligned} \right]\times100 , 0\leq{R_{sRGB} \atop\begin{aligned} G_{sRGB} \\ B_{sRGB} \end{aligned}} \leq1$ (S1)

其中

$\left[ T \right]=\left[ \begin{aligned} 0.4104 0.3576 0.1805 \\ 0.2126 0.7152 0.0722 \\ 0.0193 0.1192 0.9505 \end{aligned} \right]$ (S2)

$f\left( n \right)= \left\{ \begin{aligned} {(\frac{n+0.055}{1.055})}^{2.4}, n>0.04045 \\ \left( \frac{n}{12.92} \right), otherwise \end{aligned} \right.$ (S3)

$\left[ M_{A} \right]=\left[ \begin{aligned} \frac{X_{SW}}{X_{CW}} 0 0 \\ 0 \frac{Y_{SW}}{Y_{CW}} 0 \\ 0 0 \frac{Z_{SW}}{Z_{CW}} \end{aligned} \right]$ (S4)

On the spectrometer side: convert reflection spectral data to XYZ color gamut space

$X=k\int_{380nm}^{780nm} S\left( \lambda\right)R\left( \lambda\right)\bar{x}\left( \lambda\right)d\lambda$ (S5)

$Y=k\int_{380nm}^{780nm} S\left( \lambda\right)R\left( \lambda\right)\bar{y}\left( \lambda\right)d\lambda$ (S6)

$Z=k\int_{380nm}^{780nm} S\left( \lambda\right)R\left( \lambda\right)\bar{z}\left( \lambda\right)d\lambda$ (S7)

$k=100/\int_{380nm}^{780nm} S\left( \lambda\right)\bar{y}\left( \lambda\right)d\lambda$ (S8)

The nonlinear response of the camera can be corrected by a third-order equation, and the nonlinear response correction variable is defined as V_Non-linear_.

$V_{Non-linear}=\left[ X^{3} Y^{3} Z^{3} X^{2} Y^{2} Y^{2} X Y Z 1 \right]^{T}$ (S9)

In the dark current part of the camera, the dark current is usually a fixed value and does not change with the amount of incoming light, so a constant is given as the contribution of the dark current, and the dark current correction variable is defined as V_Dark_.

$V_{Dark}=[a]$ (S10)

Finally, VColor is used as the base, and multiplied by the nonlinear response correction of V_Non-linear_, and the result is standardized within the third order to avoid excessive correction, and finally V_Dark_ is added to obtain the variable matrix V.

$V_{Color}={[XYZ XY XZ YZ X Y Z]}^{T}$ (S11)

$V=\left[ X^{3} Y^{3} Z^{3} X^{2}Y X^{2}Z Y^{2}Z XY^{2} XZ^{2} YZ^{2} XYZ X^{2} Y^{2} Y^{2} XY XZ YZ X Y Z a \right]^{T}$ (S12)

Before using CIE DE2000 to calculate color difference, XYZ_Correct_ and XYZ_Spectrum_ must be converted from XYZ color space to lab color space. The conversion formula is as follows:

${L^{*} = 116f\left( \frac{Y}{Y_{n}} \right)-16 \atop\begin{aligned} a^{*} = 500\left[ f(\frac{X}{X_{n}})-f(\frac{Y}{Y_{n}}) \right] \\ b^{*} = 200\left[ f(\frac{Y}{Y_{n}})-f(\frac{Z}{Z_{n}}) \right] \end{aligned}}$ (S12)

$f\left( n \right)= \left\{ \begin{aligned} n^{\frac{1}{3}}, n>0.008856 \\ 7.787n+0.137931, otherwise \end{aligned} \right.$ (S13)


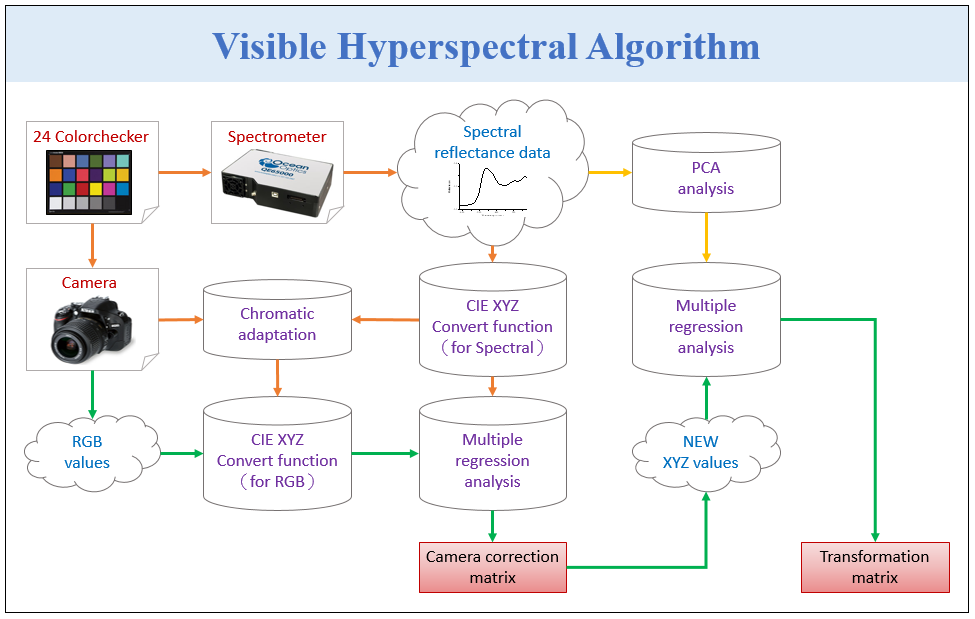


Figure S8. VIS-HSI algorithm.

**8 Experimental Environment**

**Libraries Used:**

- **YOLOv5 Implementation**: Ultralytics YOLOv5, using the official repository.
- **Programming Language**: Python 3.8
- **Deep Learning Framework**: PyTorch 1.9.0
- **Other Libraries**:
  - OpenCV for image preprocessing and augmentation
  - Albumentations for data augmentation
  - Numpy for numerical operations
  - Matplotlib for visualization
  - Scikit-learn for evaluation metrics

**Training Parameters:**

- **Batch Size**: 16
- **Learning Rate**: 0.001 (with a cosine annealing scheduler for decay)
- **Number of Epochs**: 500
- **Optimizer**: Stochastic Gradient Descent (SGD) with momentum (momentum=0.9) and weight decay (0.0005)
- **Image Size**: 640x640 pixels
- **Data Augmentation**: Random cropping, rotation, and horizontal flipping
- **Loss Function**: Binary Cross-Entropy for classification and CIoU loss for bounding box regression
- **Validation Split**: 70% training, 20% validation, 10% testing
- **Early Stopping**: Enabled after 300 epochs of no improvement in validation loss

**Hardware Environment:**

- **GPU**: NVIDIA GeForce RTX 3060 (12 GB VRAM) or AMD Radeon RX 6600 XT (8 GB VRAM)
- **CPU**: AMD Ryzen 5 5600X or Intel Core i5-11400F
- **RAM**: 32 GB DDR4
- **Storage**: 1 TB SSD
- **Operating System**: Windows 10

9. Confusion Matrix

Table S2. Confusion matrix of white light esophageal cancer imaging detection model (RGB-WLI)


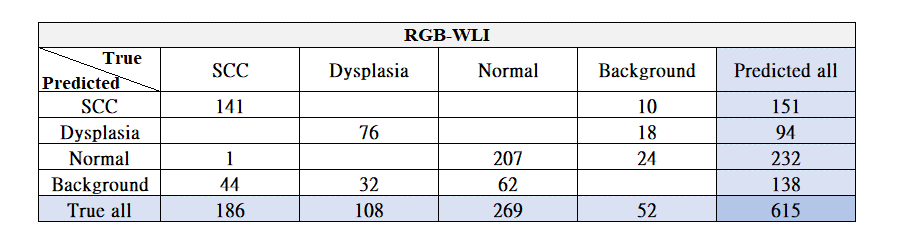


Table S3. Confusion matrix of white light hyperspectral esophageal cancer imaging detection model (HSI-WLI)

Table S4. Confusion matrix of narrow-band esophageal cancer imaging detection model (RGB-NBI)

Table S5. Confusion matrix of narrow-band hyperspectral esophageal cancer imaging detection model (HSI-NBI)

**Author Contributions:** Conceptualization, Y.-K.W, I.-C.W and H.-C.W, methodology, T.-C.M, C.-W.H and H.-C.W; software, A.M, I.-C.W and H.-C.W; validation, A.M, T.-C.M, I.-C.W and H.-C.W; formal analysis, T.-C.M, I.-C.W and H.-C.W; investigation, T.-C.M, Y.-M.T and H.-C.W; resources, Y.-K.W, C.-W.H and H.-C.W; data curation, Y.-M.T and H.-C.W; writing—Original draft preparation, A.M, Y.-M.T; writing—Review and editing, A.M, C.-W.H; supervision, Y.-K.W, C.-W.H and H.-C.W; project administration, Y.-K.W, Y.-M.T and H.-C.W. All authors have read and agreed to the published version of the manuscript.

**Funding:** This research was supported by the Ministry of Science and Technology, The Republic of China under the grants MOST 109-2314-B-037-033, 109-2622-E-194-007 and 110-2314-B-037-099. This work was financially/partially supported by the Advanced Institute of Manufacturing with High-tech Innovations (AIM-HI) and the Center for Innovative Research on Aging Society (CIRAS) from The Featured Areas Research Center Pro-gram within the frame-work of the Higher Education Sprout Project by the Ministry of Education (MOE), Kaohsiung Medical University Hospital (KMUH110-0R02), and Kaohsiung Armed Forces General Hospital research project 111-016 in Taiwan.

**Institutional Review Board Statement:** The study was conducted according to the guidelines of the Declaration of Helsinki and approved by the Institutional Review Board of Kaohsiung Medical University Hospital (KMUH) (KMUHIRB-E(II)-20190376, KMUHIRB-E(I)-20210066).

**Informed Consent Statement:** Written informed consent was waived in this study because of the retrospective, anonymized nature of study design.

**Data Availability Statement** Not Applicable

**Conflicts of Interest:** The authors declare no conflict of interest.

References

1. Redmon, J., et al. You only look once: Unified, real-time object detection. in Proceedings of the IEEE conference on computer vision and pattern recognition. 2016.

2. Liu, S., et al. Path aggregation network for instance segmentation. in Proceedings of the IEEE conference on computer vision and pattern recognition. 2018.
